# H_2_S Inhibits Hyperglycemia-Induced Intrarenal Renin-Angiotensin System Activation via Attenuation of Reactive Oxygen Species Generation

**DOI:** 10.1371/journal.pone.0074366

**Published:** 2013-09-13

**Authors:** Hong Xue, Ping Yuan, Jun Ni, Chen Li, Decui Shao, Jia Liu, Yang Shen, Zhen Wang, Li Zhou, Wei Zhang, Yu Huang, Chen Yu, Rui Wang, Limin Lu

**Affiliations:** 1 Department of Physiology and Pathophysiology, Shanghai Medical College, Fudan University, Shanghai, China; 2 Department of Pulmonary Circulation Research Center, Shanghai Pulmonary Hospital, Tongji University School of Medicine, Shanghai, China; 3 Department of Nephrology, Tongji Hospital, Tongji University School of Medicine, Shanghai, China; 4 School of Biomedical Sciences and Institute of Vascular Medicine, Chinese University of Hong Kong, Hong Kong, China; 5 Department of Biology, Lakehead University, Thunder Bay, Canada; Cedars-Sinai Medical Center, United States of America

## Abstract

Decrease in endogenous hydrogen sulfide (H_2_S) was reported to participate in the pathogenesis of diabetic nephropathy (DN). This study is aimed at exploring the relationship between the abnormalities in H_2_S metabolism, hyperglycemia-induced oxidative stress and the activation of intrarenal renin-angiotensin system (RAS). Cultured renal mesangial cells (MCs) and streptozotocin (STZ) induced diabetic rats were used for the studies. The expressions of angiotensinogen (AGT), angiotensin converting enzyme (ACE), angiotensin II (Ang II) type I receptor (AT1), transforming growth factor-β1 (TGF-β1) and collagen IV were measured by real time PCR and Western blot. Reactive oxygen species (ROS) production was assessed by fluorescent probe assays. Cell proliferation was analyzed by 5'-bromo-2'-deoxyuridine incorporation assay. Ang II concentration was measured by an enzyme immunoassay. AGT, ACE and AT1 receptor mRNA levels and Ang II concentration were increased in high glucose (HG) -treated MCs, the cell proliferation rate and the production of TGF-β1 and of collagen IV productions were also increased. The NADPH oxidase inhibitor diphenylenechloride iodonium (DPI) was able to reverse the HG-induced RAS activation and the changes in cell proliferation and collagen synthesis. Supplementation of H_2_S attenuated HG-induced elevations in ROS and RAS activation. Blockade on H_2_S biosynthesis from cystathione-γ-lyase (CSE) by DL-propargylglycine (PPG) resulted in effects similar to that of HG treatment. In STZ-induced diabetic rats, the changes in RAS were also reversed by H_2_S supplementation without affecting blood glucose concentration. These data suggested that the decrease in H_2_S under hyperglycemic condition leads to an imbalance between oxidative and reductive species. The increased oxidative species results in intrarenal RAS activation, which, in turn, contributes to the pathogenesis of renal dysfunction.

## Introduction

Diabetic nephropathy (DN) is one of the most serious complications of diabetes and is also the major cause of end stage renal failure. Recent studies show that more than 40 percent of the newly diagnosed end stage renal failure patients are DN associated [1]. DN is characterized by a progressive loss of glomerular filtration surface areas and capillary volume. The latter is largely due to an aberrant expansion, excessive production and deposition of mesangial matrix [2,3]. H_2_S, a long been recognized flammable and toxic gas, is currently accepted as another gas transmitter in the body. H_2_S has been reported for its role in the regulations of multiple functions and the abnormalities in the systemic metabolism of endogenous H_2_S involved in the pathological processes [4-6]. H_2_S generation is mainly catalyzed by three pyridoxal 5'-phosphate-dependent enzymes, cystathionine β-synthase (CBS), cystathionine γ-lyase (CSE), and 3-mercaptopyruvate sulfurtransferase (MST) [7,8]. CBS is the predominant H_2_S synthase in the central nervous system, while CSE is highly expressed in the cardiovascular system. Kidney is another major target of H_2_S in the body. CSE, CBS and MST are all identified to be expressed in kidney. H_2_S participates in the regulation of renal functions. It increases urinary sodium excretion via both vascular and tubular actions in the kidney [9]. In 5/6 nephrectomy-induced chronic renal dysfunction rats, the capacity of endogenous H_2_S production was down-regulated [10]. In 2008, Tripatara P et al reported that H_2_S donor NaHS attenuated ischemia/reperfusion induced renal injury, and the endogenous H_2_S produced by CSE was essential for protective effects [11]. In our previous study, we noticed that the H_2_S generation was reduced in the early stage of diabetes in rats, and the decrease in H_2_S production was associated with an increase in reactive oxygen species (ROS) [12]. Recently, Ahmad FU observed that NaHS lowers blood pressure and attenuates diabetes-induced renal damage in spontaneous hypertension rats [13]. Yamamoto J also reported that CSE expression was markedly reduced under diabetic conditions, whereas CBS expression was unaffected [14]. In cultured renal vasculature, Sen U et al reported that CBS, CSE and 3-MST triple gene transfections protect homocysteine-induced vascular injury, and the effect was combined with an increase in H_2_S generation [15]. The intravenous injection of H_2_S donor attenuated the tissue injury in renal ischemia/reperfusion porcine model, and the H_2_S supplementation was associated with the suppressions of oxidative stress, inflammation and nitrosative stress [16]. However, Francescato HD et al recently reported that the inhibition of H_2_S production by CSE inhibitor DL-propargylglycine (PPG) protected the gentamicin-induced kidney injury [17].

The abnormality in intrarenal renin-angiotensin system (RAS) plays a crucial role in the onset and development of DN. The over activation in intrarenal RAS has been reported to be associated with glomerular enlargement and secondary glomerulosclerosis, tubular epithelial to mesenchymal transition, interstitial fibroblast proliferation and extracellular matrix (ECM) deposition [18,19]. Currently, the inhibitors of RAS, including angiotensin converting enzyme inhibitor (ACEI) or angiotensin II (AngII) type I receptor (AT1) blockers (ARB), have been used as first line medication in the clinical treatment of DN. Data suggest that there is an interaction between ROS overproduction and RAS activation [20-22]. However, the exact mechanism has not been fully elucidated. Recently, Lu M et al reported that NaHS treatment inhibits elevation in renin activity and blunted blood pressure increase in 2-kidney 1-clip hypertensive rats [23]. This experiment is aimed at investigating the effects of H_2_S production suppression under hyperglycemic condition on ROS production and intrarenal RAS activation.

## Materials and Methods

### Materials and reagents

Dulbecco’s Modified Eagle’s Medium (DMEM), fetal bovine serum (FBS), streptozotocin (STZ), D-glucose, mannitol, DL-propargylglycine (PPG), diphenylenechloride iodonium (DPI), and NaHS were purchased from Sigma (St Louis, MO). NaHS was used as a H_2_S donor, which was also widely employed in previous studies [24-26]. Antibody against collagen IV was purchased from Santa Cruz Biotechnology Inc. (Santa Cruz, CA). SYBR Green QRT-PCR Master Mixture was from Applied Biosystems (Tokyo, Japan). RNA extraction kit was from Sangon Co. (Shanghai, China). Polyvinylidene difluoride (PVDF) membrane was from Amersham (Piscataway, NJ). Kodak X-Omat K film was from Kodak Co. (Xiamen, China). Enhanced chemiluminescent detection kit (ECL detection kit) was from Pierce Biotechnology Inc. (Rockford, IL). 5’-Bromo- 2’-deoxyuridine (BrdU) incorporation kit was from Roche (Mannheim, Germany). ROS assay kit was from Beyotime (Jiangsu, China). All other reagents used in this study were of analytical grade.

### Animal model

Age-matched male SD rats, weighing 180–210 g, were provided by the Shanghai Laboratory Animal Center. All procedures abided by the Criteria of the Medical Laboratory Animal administrative Committee of Shanghai and the Guide for Care and Use of Laboratory Animals of Fudan University, and were approved by the Ethics Committee for Experimental Research, Shanghai Medical College, Fudan University. The rats were held for 3-days acclimatization before use and had free access to water and standard chow. Diabetes was induced by a single intraperitoneal (i.p.) injection of STZ (65 mg/kg) dissolved in 0.1 M sodium citrate buffer (pH 4.0). Only those rats with plasma glucose concentrations >16.7 mM 1 week after STZ injection were recruited in the study. The rats were randomly divided into four groups (n = 6 for each group): (i) control (C) rats, injected with 0.1 M sodium citrate buffer, pH 4.0; (ii) diabetic (D) rat, injected with STZ (65 mg/kg); (iii) diabetic rats with injection of NaHS (D + NaHS) and (iv) non-diabetic rats with injection of NaHS (C + NaHS) (50 μmol/kg/day, i.p.) during the third week. At the end of the third week, rats were sacrificed and the plasma and renal tissues were harvested and stored at −80 °C until use.

### Measurement of blood glucose

The glucose level in the blood sample collected from the abdominal aorta was measured using blood glucose detection kit assays (Jiancheng Bioengineering Company, Nanjing, China).

### Cell culture

The rat glomerular MC line (HBZY-1) was purchased from China Center for Type Culture Collection (Wuhan, China) and cultured in normal DMEM media (5.5 mM D-glucose) supplemented with 10% FBS in an atmosphere of 5% CO_2_ at 37 °C. High glucose (HG) culture media was made by supplementing the normal DMEM media with additional D-glucose for a final D-glucose concentration at 25 mM. The osmotic control media was made by supplementing normal media with 19.5 mM mannitol.

### Isolation of total RNA and synthesis of cDNA

Total RNA of cultured rat MCs or renal cortex was isolated according to the protocol of RNA extraction kit. The amount of RNA isolated was determined by measuring the specific absorbance at 260 nm. One microgram of total RNA was used for cDNA synthesis in a 20 µl reaction mixture that contained 1 µg oligo dT, 10 mM dNTP, 20 U RNase inhibitor and 200 U M-MLV reverse transcriptase. A 1-µl aliquot of the resulting single-strand cDNA was used for polymerase chain reaction (PCR).

### Quantitative real-time PCR

SYBR Green qRT-PCR was used to quantify the relative abundance of target messenger RNA (mRNA) in the samples. The accumulated fluorescence was detected using the iCycler iQ PCR detection system (Bio-Rad, Hercules, CA). The PCR amplification conditions were as follows: pre-denaturing at 95 °C for 3 min, followed by 40 cycles of amplifications by denaturing at 95 °C for 30 s, annealing at 62 °C (for TGF-β1), or 60 °C (for angiotensinogen (AGT), ACE, AT1 receptor, collagen IV and glyceraldehyde 3-phosphate dehydrogenase (GAPDH)) for 1 min, extension at 72 °C for 1 min. After a final extension at 72 °C for 10 min, the amplified products were subjected to a stepwise increase in temperature from 55 to 95 °C to construct dissociation curves. The relative amount of each mRNA was normalized to a housekeeping gene, GAPDH. Each sample was run and analyzed in triplicate. The average of the relative amount of each mRNA in control group is defined as 1.0. All PCR primer sequence and product characteristics are listed in Table 1.

**Table 1:** PCR primer pairs and product characteristics.

Target		Oligonucleotide sequence	Tm °C	Product size (bp)
AT1	F	5’-CTC AAG CCT GTC TAC GAA AAT GAG-3’	60	204
	R	5’-TAG ATC CTG AGG CAG GGT GAA T-3’		
AGT	F	5’-GCA AAA ATC ATG GCC TTC ACC C-3’	60	128
	R	5’-AAA CAA ACC CAC ACC CCA GGA G-3’		
Collagen IV	F	5’-ATT CCT TTG TGA TGC ACA CCA G-3’	60	151
	R	5’-AAG CTG TAA GCA TTC GCG TAG TA-3’		
ACE	F	5’-GTG TTG TGG AAC GAA TAC GC-3’	60	187
	R	5’-CCT TCT TTA TGA TCC GCT TGA-3’		
TGF-β1	F	5’-TGG CGT TAC CTT GGT AAC C-3’	62	277
	R	5’-GGT GTT GAG CCC TTT CCA G-3’		
CSE	F	5’-GAC GAG GAA TTG CTT GGA AA-3’	60	180
	R	5’-GAT GCC ACC CTC CTG AAG TA-3’		
GAPDH	F	5’-CCC TTC ATT GAC CTC AAC TAC ATG-3’	60	216
	R	5’-CTT CTC CAT GGT GGT GAA GAC-3’		

F, forward Primer; R, reverse primer; Tm, melting temperature.

### Measurement of Ang II by enzyme immunoassay

Ang II levels in the conditioned supernatants were assayed using a commercially available enzyme immunoassay-based colormetric kit (Phoenix Pharmaceuticals, Burlingame, CA, USA). The supernatants were collected and passed through a Centricon-10 column with a cutoff of >10,000 Da (Amicon, Beverly, MA). The filtrate was used for Ang II measurements following the manufacturer’s protocol. Absorbance was measured at 450 nm using a TECAN Infinite M200 microplate reader (Salzburg-Umgebung, Salzburg, Australia), and the concentrations of Ang II in samples were determined by extrapolation from a standard curve.

### Cell proliferation analysis

A total of 10^3^ cells/well were cultured in 96-well plates in 10% FBS-supplemented media. After 24 h of sub-culturing, the cells were serum starved for 24 h, and then treated with normal DMEM media, high glucose culture media, 19.5 mM mannitol, losartan with normal DMEM media, losartan with high glucose culture media, DPI with normal DMEM media, or DPI with high glucose culture media. In co-treatment experiments, losartan or DPI was added 30 min prior to high glucose culture media treatment. After 48 h, cell proliferation was assessed by BrdU incorporation assays as previously described [9]. Briefly, 1 µM BrdU labeling solution was added into the medium. After incubating the cells for an additional 4 h at 37 °C, the cells were fixed, denatured and then incubated in anti-BrdU-POD (peroxidase) antibody for another 90 min at room temperature. At the end of the incubation, the cells were rinsed with PBS 3 times to remove excessive antibody; then, 100 µl of substrate solution was added into each well. After another incubation period for 30 min at room temperature, the absorbance of the samples was measured on a TECAN Infinite M200 microplate reader (Salzburg-Umgebung, Salzburg, Australia) at 370 nm, while the absorbance obtained at 492 nm served as a reference value.

### Western blot analysis

For media of cultured renal MCs, proteins with molecular weights above 30 kD were separated by Amicon Ultra 4 [12]. Then, 30 µg of protein sample was electrophoresed on an 8% polyacrylamide SDS gel and transblotted onto a PVDF membrane at 270 mA for 90 min. The membranes were blocked with 5% skim milk in Tris-buffered saline (TBS) and 0.1% Tween (TBS/Tween) for 1 h at room temperature with gentle rocking and followed by incubation in goat anti-rat collagen IV antibodies (1:2000) at 4 °C overnight. After three washes with TBS/Tween, the membranes were incubated with secondary anti-goat antibody (1:2000) for 1 h at room temperature. The hybridizing signals were developed using the ECL detection kit according to the manufacturer’s instructions and exposed to X-ray film. The relative intensity of the bands exposed on the films was quantified using Smart viewer software (Furi Technology Co, Shanghai, China).

### Measurement of ROS production

Experiments were performed using the ROS assay kit (Beyotime, Haimen, China) according to the manufacturer’s instructions. In brief, cells seeded in 96-well plates were incubated with 10 mM 2’, 7’-Dichlorofluorescein diacetate (DCFH-DA) fluorescent probes (100 μl/well) at 37 °C for 30 min and then washed with PBS three times in order to remove residual probes. The fluorescence intensity at 488 nm excitation wavelength and 525 nm emission wavelength was measured using a luminometer (Tecan, Salzburg, Austria).

### Statistical analysis

Data are expressed as means ± standard error (SE) of the mean for the repeats of individual experiments indicated. The data were analyzed by one-way ANOVA using Student-Newman-Keuls test. A *P* value of less than 0.05 was considered statistically significance.

## Results

### Effect of HG treatment on the local RAS in renal MCs

**Figure 1:**
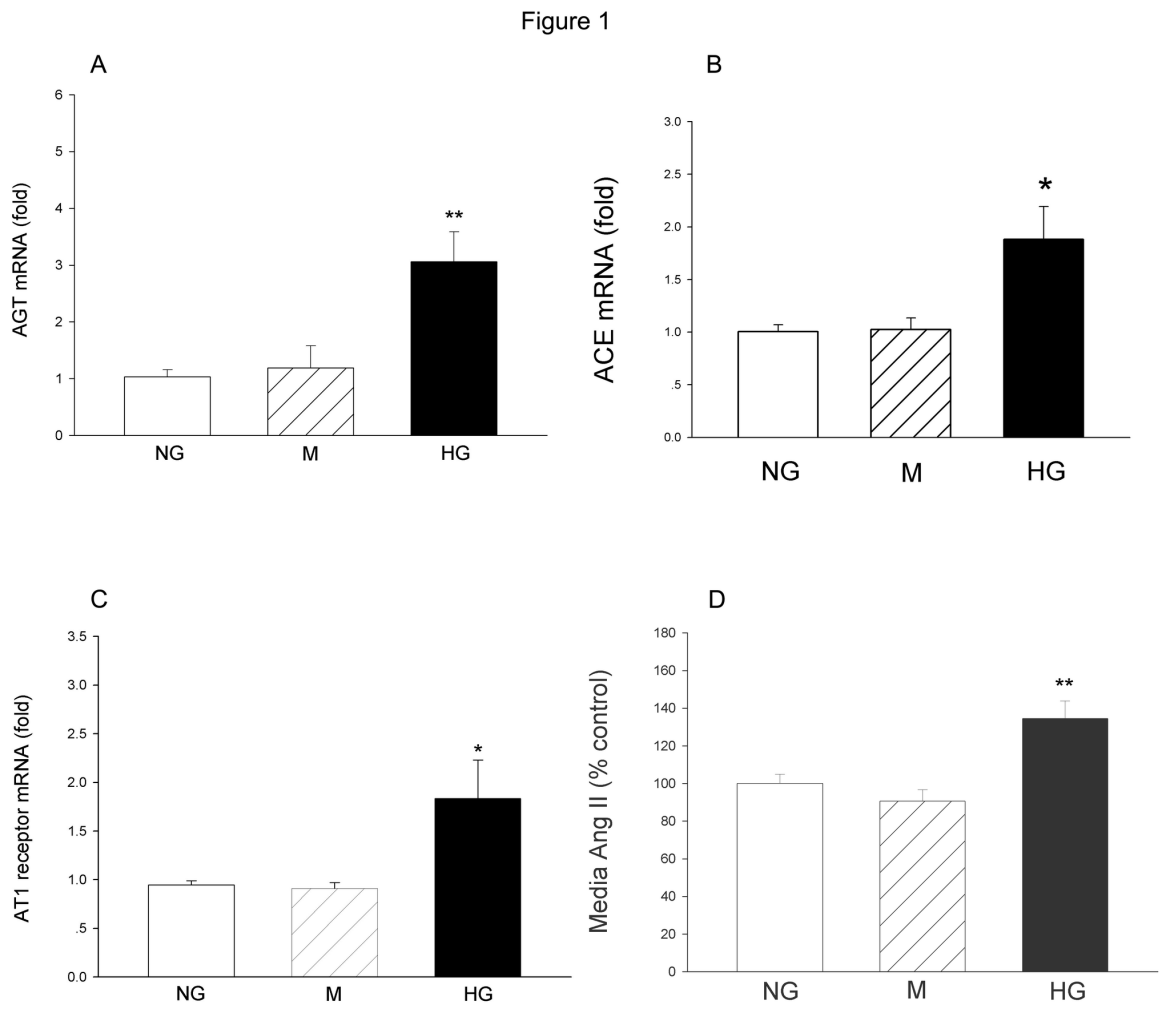
The changes in RAS components in the cultured renal MCs after HG treatment. The mRNA levels of AGT (A), ACE (B) and AT1 receptor (C) were measured by real-time PCR. The Ang II concentrations in the medium were measured by enzyme immunoassay. The data were expressed as Mean±SE. *P<0.05 versus NG group; **P<0.01 versus NG group, n=5.

Culturing the cells in HG for 24 h, real-time PCR result showed that AGT, ACE and AT1 receptor mRNA levels were significantly increased when compared with that of normal glucose (NG)-treated cells. Meanwhile, enzyme-linked-immunosorbent serologic assay (ELISA) result showed that the Ang II concentration in HG media was elevated. In contrast, osmotic control mannitol did not show significantly influence on AGT, ACE and AT1 receptor mRNA levels, as well as the Ang II concentration in the media when compared to that of NG group (Figure 1).

### Effect of AT1 receptor blockade on HG-induced cell proliferation, TGF-β1 and collagen IV expression

**Figure 2:**
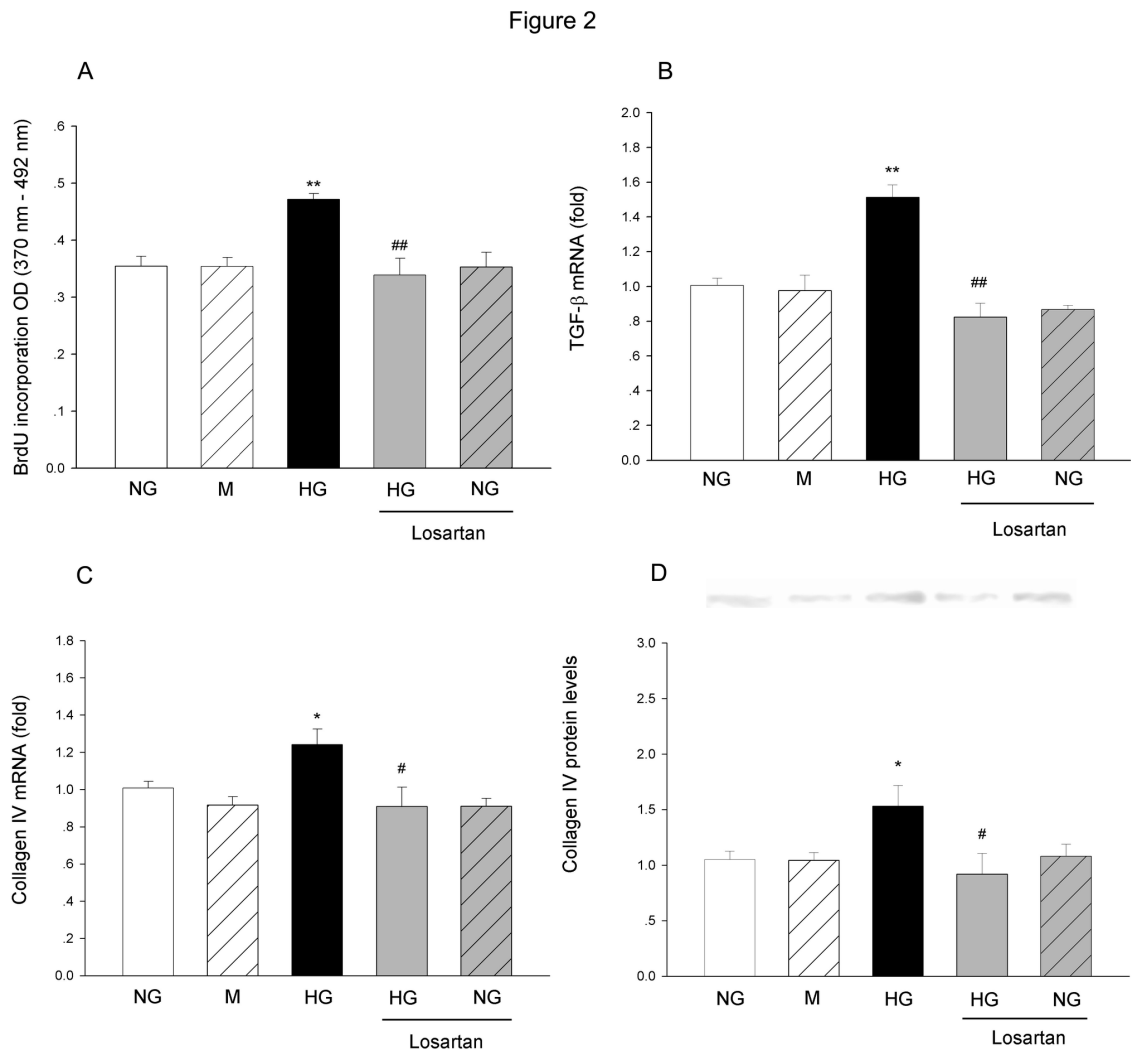
The effects of losartan on HG-induced changes in MCs proliferation, TGF-β1 and collagen IV expression. (A) The cell proliferation was measured by BrdU incorporation assay at 48h after HG treatment with or without losartan (1 µM). TGF-β1 (B) and Collagen IV (C) mRNA levels were measured by real-time PCR at 24h after HG treatment. Collagen IV protein levels (D) were measured by Western blot. The data were expressed as Mean±SE. *P<0.05 versus NG group; **P<0.01 versus NG group; ^#^ *P*<0.05 versus HG group; ^# #^ *P*<0.01 versus HG group, n=5.

After culturing the cells in HG media for 48 h, the BrdU incorporation assay result showed that the cell proliferation rate was increased significantly when compared with the NG-treated cells. Mannitol did not show obvious influence on the cell proliferation. Real-time PCR results showed that TGF-β1 and collagen IV mRNA levels were increased significantly in HG-treated groups. Western blot results also showed a significant increase in collagen IV protein level. Mannitol did not show obvious influence on TGF-β1 and collagen IV mRNA levels, or collagen IV protein level. Blockade on AT1 receptor by losartan (1 µM) abolished all the changes induced by HG treatment, including increase in cell proliferation, elevations in TGF-β1 and collagen IV mRNA level, and collagen IV protein level. Application of losartan did not show obvious influence on cell proliferation, TGF-β1 and collagen synthesis in NG-treated cells (Figure 2).

### Effect of NADPH oxidase inhibition on HG-induced cell proliferation, TGF-β1 and collagen IV expression

**Figure 3:**
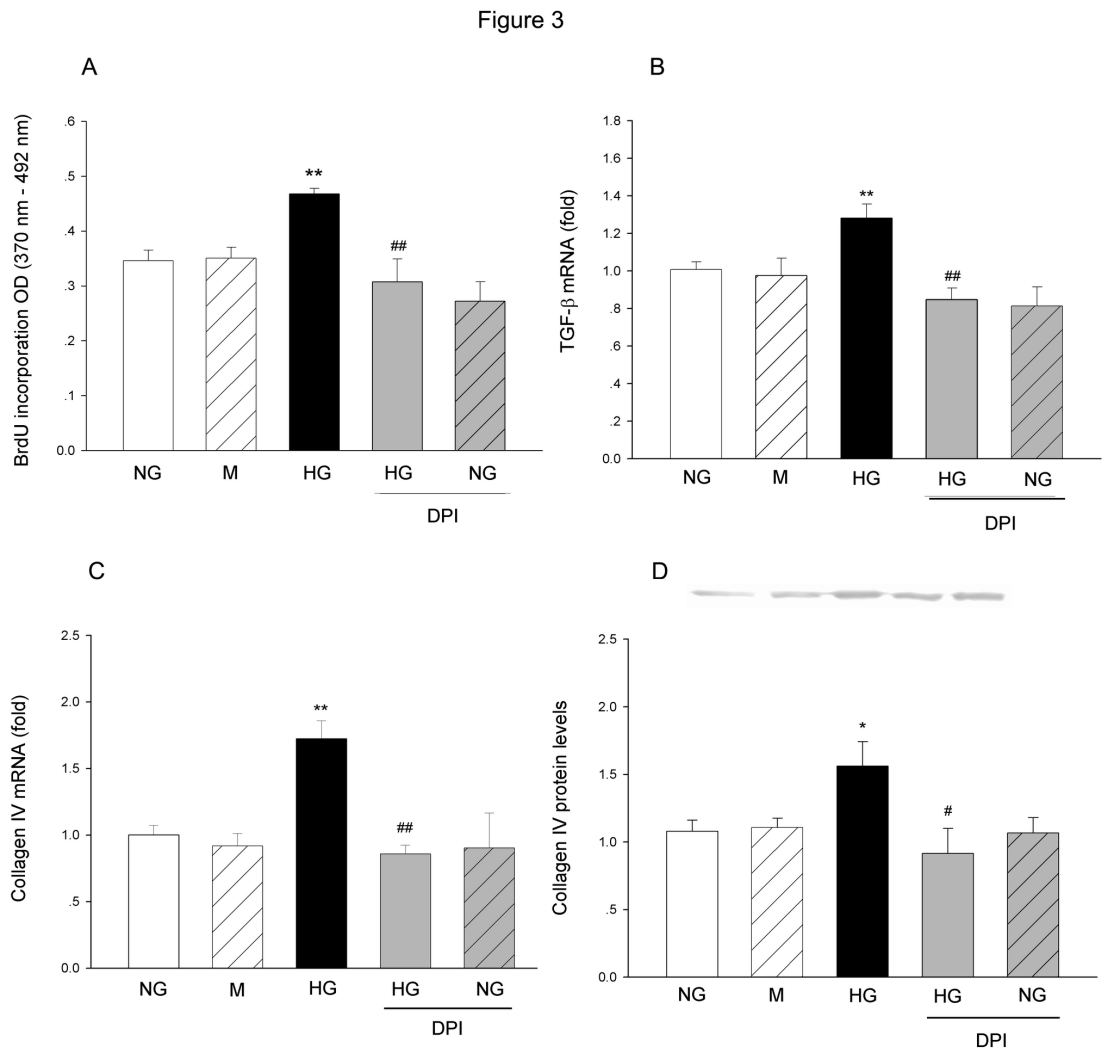
The effects of DPI on HG-induced changes in MCs proliferation, TGF-β1 and collagen IV expression. (A) The cell proliferation was measured by BrdU incorporation assay at 48h after HG treatment with or without DPI (1 µM). The TGF-β1 (B) and Collagen IV (C) mRNA levels were measured by real-time PCR at 24h after HG treatment. The collagen IV protein levels (D) were measured by Western blot. The data were expressed as Mean±SE. *P<0.05 versus NG group; **P<0.01 versus NG group; ^#^ *P*<0.05 versus HG group; ^# #^ *P*<0.01 versus HG group, n=5.

Following cell culture in HG media for 48 h, the cell proliferation rate, TGF-β1 and collagen IV mRNA levels, and collagen IV protein level were significantly increased in HG-treated groups. Mannitol did not show obvious influence on any of them. Blockade of nicotinamide adenine dinucleotide phosphate (NADPH) oxidase by DPI abolished the changes induced by HG treatment, including the increase in cell proliferation, elevations in TGF-β1 and collagen IV mRNA level, and increase in collagen IV protein level. Application of DPI did not show significant effect on NG-treated group (Figure 3).

### Effect of NaHS, DPI and Apocynin on HG-induced ROS generation

**Figure 4:**
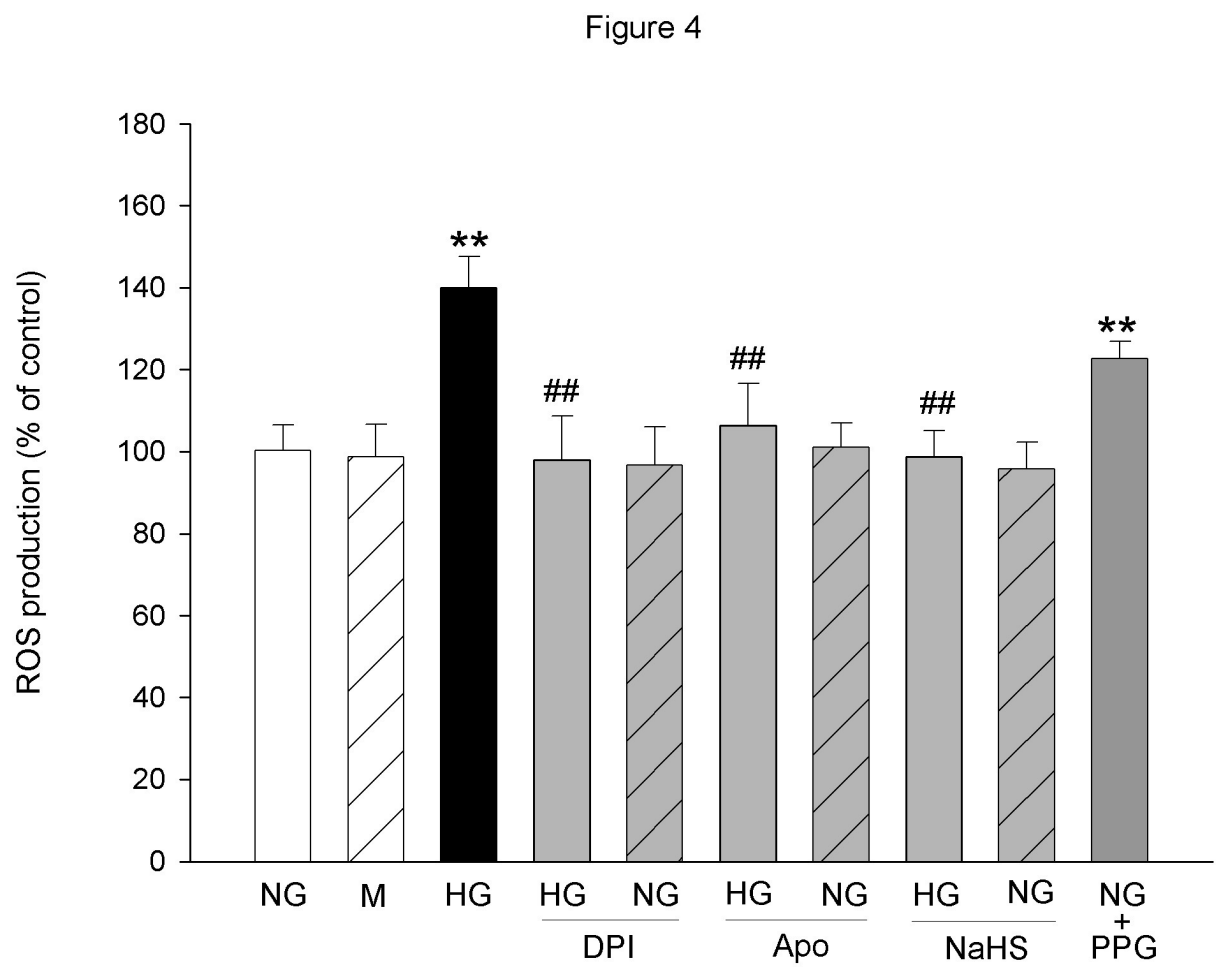
The effects of NaHS, PPG, DPI or Apocynin on ROS production in the cultured MCs. The ROS levels were measured by DCFH-DA fluorescent probe assay at 24 h after treatment. The data were expressed as Mean±SE. **P<0.01 versus NG group; ^# #^ *P*<0.01 versus HG group, n=8.

DCFH-DA fluorescent probe assay results showed that culturing the cells in HG media for 24 h resulted in a significant increase in ROS generation compared to NG-treated cells. Mannitol did not display obvious influence on ROS generation. Application of NADPH oxidase inhibitor DPI (1 µM) or apocynin (10 µM) abolished the HG-induced increase in ROS generation. Neither DPI nor apocynin alone showed obvious influence on the ROS generation in NG-cultured cells. NaHS (30 µM), a donor of H_2_S, abolished the increase in ROS generation induced by HG treatment as well. NaHS alone did not influence the ROS generation in the NG-cultured cells significantly. PPG (1 mM), an endogenous H_2_S synthesizing enzyme CSE inhibitor, elevated the ROS generation significantly in the NG-cultured cells (Figure 4).

### Effect of NaHS or DPI on HG-induced changes in AGT, ACE and AT1 receptor expressions

**Figure 5:**
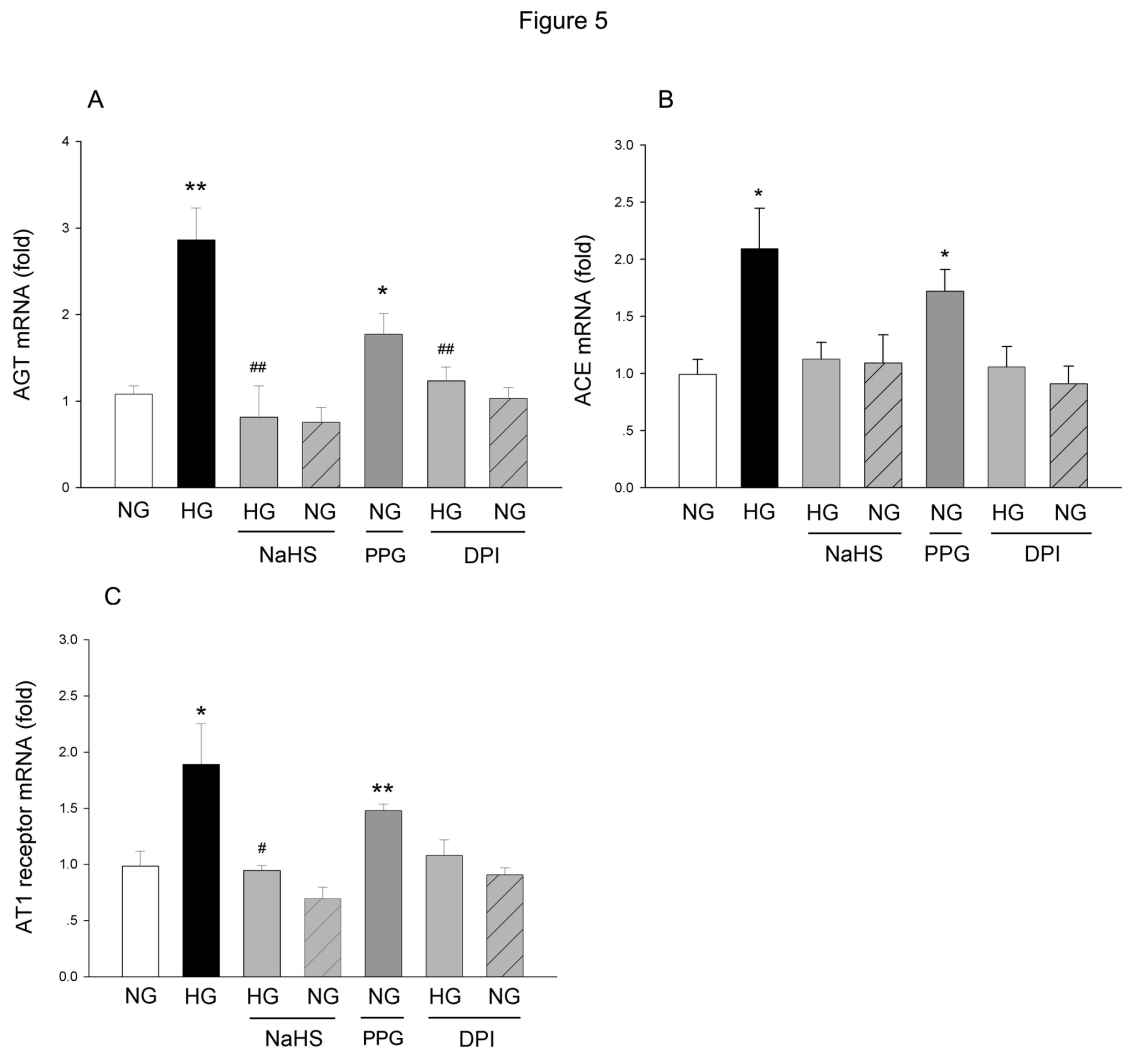
The effects of NaHS, PPG or DPI on HG-induced changes in AGT, ACE and AT1 receptor mRNA levels in the cultured MCs. The mRNA levels were measured by real-time PCR at 24h after HG treatment. The data were expressed as Mean±SE. *P<0.05 versus NG group; **P<0.01 versus NG group; ^#^ *P*<0.05 versus HG group; ^# #^ *P*<0.01 versus HG group, n=5.

As shown in Figure 5A, the AGT mRNA level was increased significantly after culturing the cells in HG for 24 h. Application of NaHS abolished the elevation in AGT mRNA level. NaHS did not affect the AGT mRNA level significantly in NG-cultured cells. Similar result was observed from the application of DPI, which abolished the elevation in AGT mRNA induced by HG treatment. DPI did not showed obvious influence on AGT mRNA level in the NG-cultured cells. Application of PPG increased AGT mRNA level significantly in the NG-cultured cells. The changes in ACE (Figure 5B) and AT1 (Figure 5C) mRNA levels showed patterns similar to that of AGT.

### Effect of NaHS application on animal blood glucose and AGT, ACE and AT1 receptor mRNA levels in diabetic rat kidney

**Figure 6:**
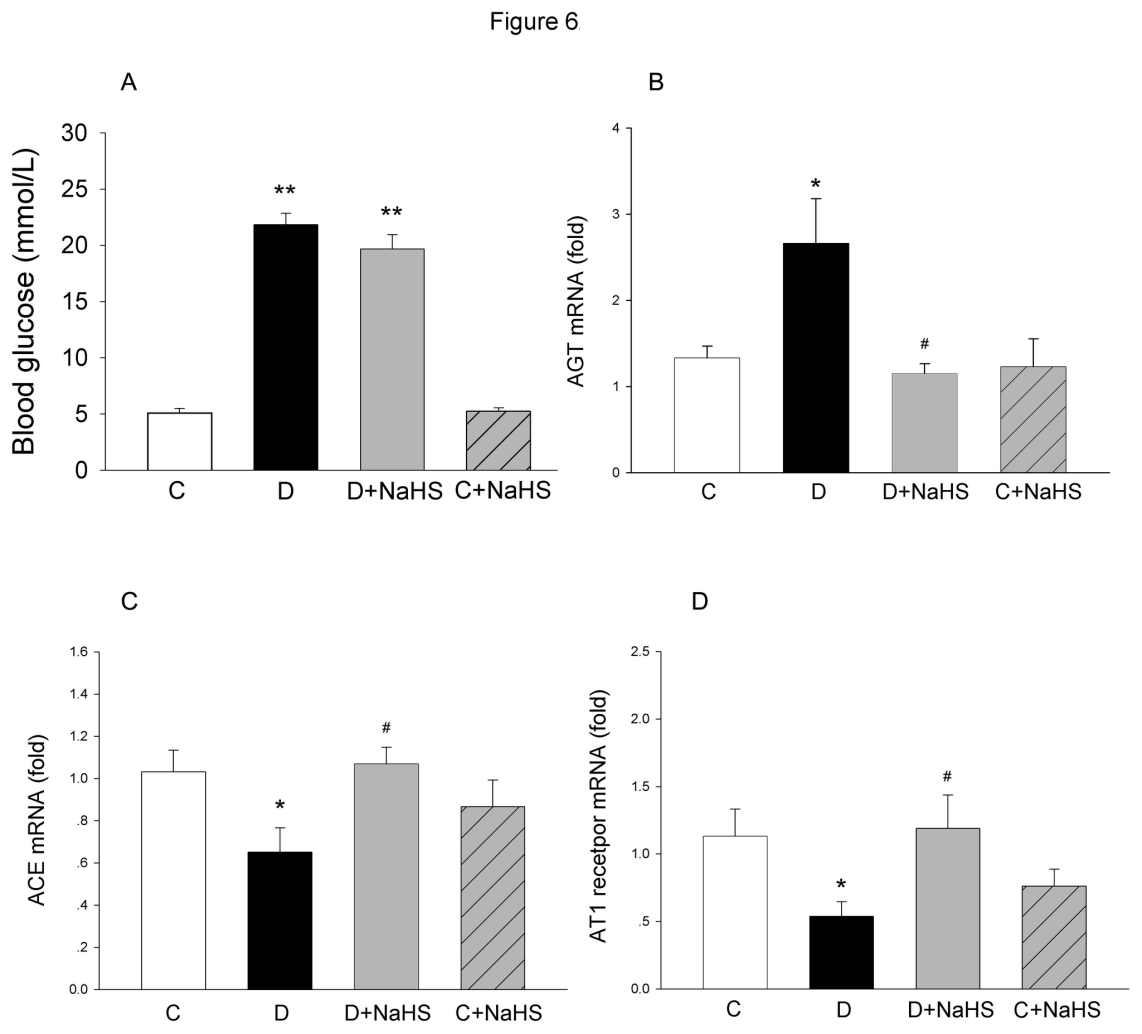
Measurement of blood glucose, AGT, ACE and AT1 receptor mRNA levels in animals of control (C), diabetic (D), diabetic treated with NaHS (D+NaHS) and control treated with NaHS (C+NaHS) rats. The blood glucose (A) was measured by glucose detection kit, and the mRNA levels for AGT (B), ACE (C), and AT1 receptor (D) were measured by real-time PCR 3 weeks after STZ injection. The data were expressed as Mean±SE. *P<0.05 versus control rats; ^#^ *P*<0.05 versus diabetic rats, n=6.

The blood glucose was increased significantly in STZ-induced diabetic rats. NaHS treatment did not show obvious effects on the blood glucose concentrations in both diabetic and non-diabetic animals (Figure 6A). Real-time PCR result showed that the AGT mRNA level was elevated significantly in the kidneys of diabetic rats. NaHS treatment (50 µmol/kg.d) reversed the change in AGT mRNA level. However, NaHS did not show any obvious influence on the AGT mRNA level in the kidneys of non-diabetic rats (Figure 6B). Both ACE and AT1 receptor mRNA levels were decrease obviously in the kidneys of diabetic rats. NaHS treatment abolished the decreases in both ACE and AT1 mRNA levels in diabetic rats, but did not show obvious influences in non-diabetic rats (Figure 6C and 6D).

### Effect of Ang II on CSE and CBS expression in cultured rat renal MCs

**Figure 7:**
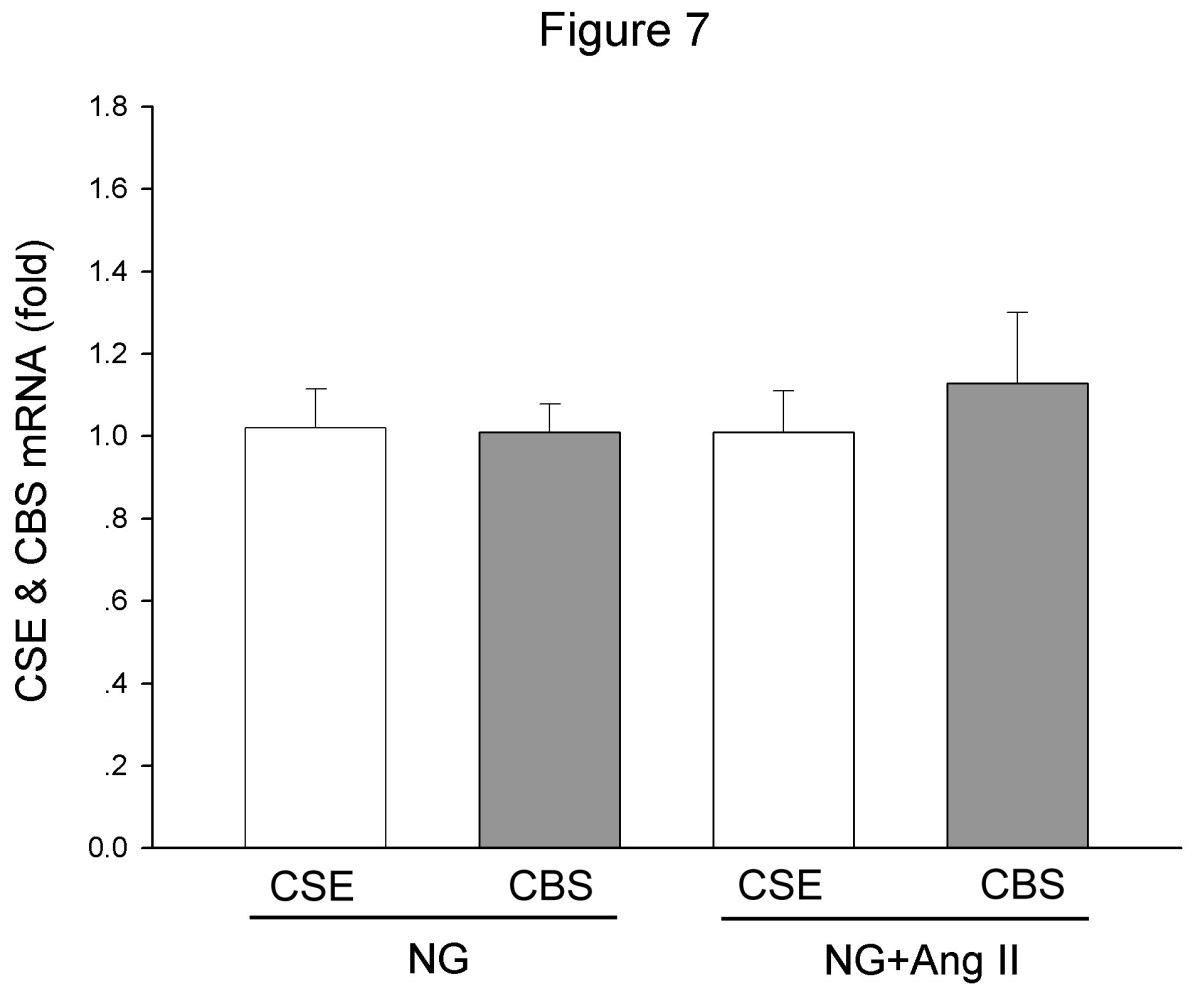
The effects of Ang II on CSE or CBS expression in the cultured MCs. The CSE and CBS mRNA levels were measured by real-time PCR at 24h after Ang II (10 nM) treatment. The data were expressed as Mean±SE, n=6.

Real-time PCR result showed that application of Ang II (10 nM) did not produce an obvious effect on both CSE and CBS mRNA (Figure 7).

## Discussion

Over activation of intrarenal RAS is recognized as a crucial factor in the pathogenesis of DN. Ang II stimulates the diabetic-induced tubular epithelial cell to mesenchymal myofibroblast transition, interstitial fibroblast proliferation, and ECM production [27]. In renal glomerulus, the activation in RAS may result in apoptosis and the loss of endothelial cells and podocytes, but can also result in proliferation of MCs [28-30]. MCs are important in maintaining the normal morphology and functions of glomeruli. Ordinarily, MCs produce small amount of ECM proteins, including fibronectin and collagen type I, IV etc, to substitute the metabolized ECM. Under pathological conditions, like diabetes, both the cell proliferation rate and ECM production were increased, and those changes will finally contribute to the thickening of glomerular basal membrane, and glomerular ECM expansion.

The renal MC is an important target of Ang II and the expression of all elements in RAS [19]. Ang II has been proved to stimulate ECM synthesis by MCs [31]. In the cultured MCs, the result showed that local RAS was activated under high glucose condition. The expressions of AGT, ACE and AT1 receptor were increased, and Ang II concentration in the culture media was elevated. The over activation of RAS was associated with the functional changes of MCs, including stimulating cell proliferation, TGF-β and ECM productions. AT1 receptor blockade reversed the HG-induced cell proliferation and ECM productions. These results are consistent with the previous observations [32,33].

Meanwhile, the ROS production was also elevated under high glucose condition. Oxidative stress has been noticed as a pathogenic factor for DN. In 2002, Catherwood MA et al report that high glucose stimulated ROS production in cultured renal mesangial cells, and the oxidative stress enhanced fibronectin and collagen IV synthesis [34].

In the experiment, DPI, a NADPH oxidase inhibitor, blocked the increase of ROS. This result suggested that the increase of ROS is mainly achieved through the NADPH oxidase pathway. Application of DPI attenuated HG-induced cell proliferation and ECM production suggested that HG-induced ROS increase produced a similar result to RAS over activation.

It has been noticed that there is interaction between RAS and ROS. Ang II stimulates ROS production by activating AT1 receptor. This phenomenon has been noticed in our previous studies as well as many other studies in cardiovascular and nervous fields [35-37]. ROS is even considered as a downstream signaling molecule of Ang II. In this experiment, the data showed the elevations of ROS and RAS were closely related. However, it seems that the changes of ROS was upstream to of RAS, since the blockade on ROS increase by application of DPI dismissed the HG-induced upregulations in RAS elements expressions, as well as the cell proliferation and ECM production in MCs.

In the experiment, we also provided evidence that suggests the supplementation of H_2_S induced similar effects of DPI, which attenuated HG-induced ROS production, and RAS over activation. On the other hand, the inhibition of endogenous H_2_S production by PPG, a CSE inhibitor, induced similar effects on HG treatment, including elevation of ROS level, and upregulating of AGT, ACE and AT1 expressions.

H_2_S is a reductive gas molecule. In Fujita K’ s study, H_2_S was proved to have a direct effect on scavenging ROS [38]. However, it had been reported that H_2_S inhibited ROS formation via different pathways. In Muzaffar S’s study, the long-term protecting effect of H_2_S on oxidative stress is related to suppressing NADPH oxidase [39]. In Zhong X’ s study, exogenous H_2_S (NaHS) down-regulated the expression of mitochondrial NOX4, and decreased the levels of ROS [40]. In Suzuki’s study, hyperglycemia resulted in a switch from oxidative phosphorylation to glycolysis, which was partially corrected by H_2_S supplementation [41]. Si YF reported that treatment with H_2_S not only improved mitochondrial function and reduced mitochondrial ROS formation, but also suppressed NOX2 and gp47 phox expression [42]. In the study, the data showed that the decrease in H_2_S induced the imbalance between oxidative and reductive species, which resulted in the intrarenal RAS up-regulation. Restoration of the balance can be accomplished through inhibiting ROS production or supplementing H_2_S to prevent RAS activation.

Oxidative stress may appear at a very early stage of DN. In cultured cell, the ROS production increased within an hour. Thus we speculate that high glucose stimulation shifts the balance between oxidative and reductive species; the excessive ROS activates the local RAS and the increase in Ang II stimulates more ROS production via activation of AT1 receptor. Such pathological vicious cycle, in turn, leads to initiation of MCs proliferation and elevated ECM production.

The observations on the animals were not completely consistent with the footprint of the cell study. In diabetic rats, the AGT expression was increased, while the expressions of ACE and AT1 receptor were down-regulated. These changes were consistent with the previous study in vivo, including the data from clinical studies [27,43-45]. There is no clear answer for the different between in vitro and in vivo studies, but the inconsistency is not unique to RAS observation. In the experiment, the supplementation of H_2_S restored the elevation of AGT, as well as the reductions in ACE and AT1 expressions. H_2_S supplementation did not influence the plasma Ang II level, which suggested that the effect of H_2_S is not achieved by manipulating glucose level. From the result that Ang II did not influence the expression of both CSE and CBS, we speculate that the effect of H_2_S on regulating RAS component was mediated by manipulating oxidative state.

In summary, hyperglycemia or high glucose suppresses endogenous H_2_S production which leads to an imbalance between oxidative and reductive species. The redundancy in oxidative species results in over activation of the intrarenal RAS, which in turn leads to MC proliferation and excessive ECM productions. Since the over activation of RAS can stimulate more ROS production, the changes in H_2_S may initiate a vicious cycle that will lead to phathological changes in the kidney. Restoring the harmonization between oxidative and reductive species by either NADPH oxidase inhibition or H_2_S supplementation at early stage of diabetes may benefit the attempt to cease the vicious cycle.
